# Reprogramming cell fates by small molecules

**DOI:** 10.1007/s13238-016-0362-6

**Published:** 2017-02-17

**Authors:** Xiaojie Ma, Linghao Kong, Saiyong Zhu

**Affiliations:** Life Sciences Institute, Zhejiang University, Hangzhou, 310058 China

**Keywords:** reprogramming, small molecules, stem cells, cell fates

## Abstract

Reprogramming cell fates towards pluripotent stem cells and other cell types has revolutionized our understanding of cellular plasticity. During the last decade, transcription factors and microRNAs have become powerful reprogramming factors for modulating cell fates. Recently, many efforts are focused on reprogramming cell fates by non-viral and non-integrating chemical approaches. Small molecules not only are useful in generating desired cell types *in vitro* for various applications, such as disease modeling and cell-based transplantation, but also hold great promise to be further developed as drugs to stimulate patients’ endogenous cells to repair and regenerate *in vivo*. Here we will focus on chemical approaches for generating induced pluripotent stem cells, neurons, cardiomyocytes, hepatocytes and pancreatic β cells. Significantly, the rapid and exciting advances in cellular reprogramming by small molecules will help us to achieve the long-term goal of curing devastating diseases, injuries, cancers and aging.

## INTRODUCTION

Studies of developmental biology have demonstrated that the zygote is totipotent and can gradually differentiate into all tissues and organs of the human body, that is composed by more than 200 types of specialized cells. However, the demonstration of interchanging cell fates and converting one cell type into another by different strategies, such as nuclear transfer or cell fusion, has challenged the traditional cognition of development and regeneration (Gurdon, 1962; Tada et al., 1997). In 2006, Yamanaka and Takahashi reported the development of the induced pluripotent stem cell (iPSC) technology, which can convert somatic cells to iPSCs using four transcription factors (TFs) (Takahashi and Yamanaka, 2006). During the last 10 years, iPSC technology has provided many fundamental insights into the molecular mechanisms of cell fate transition, and already demonstrated promising results in various applications, including disease modeling, drug screening, and cell-based therapy (Takahashi and Yamanaka, 2016). Recently, different combinations of lineage-specific TFs have been screened and applied to generate various cell types, including neurons, cardiomyocytes, hepatocytes, and pancreatic β cells (Huang et al., 2011; Ieda et al., 2010; Vierbuchen et al., 2010; Zhou et al., 2008). However, TF-based reprogramming approaches face many challenges in efficiency, safety, and *in vivo* delivery. As a novel and promising solution, small molecules are easy to apply and remove, more efficient and amenable to scale up. Small molecules not only are useful in generating desired cell types *in vitro* for various applications, but also can be further developed as drugs to stimulate patients’ endogenous cells to repair and regenerate *in vivo*.

**Figure 1:**
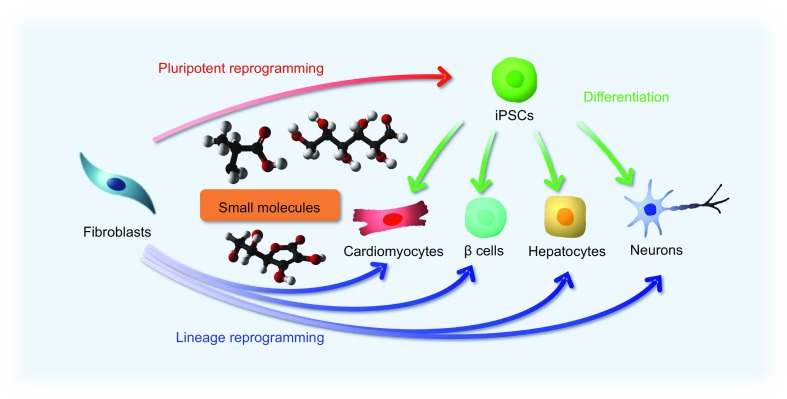
**Reprogramming cell fates by small molecules**. Chemical approaches can be widely applied to manipulate cell fates and states, including pluripotent reprogramming, directed differentiation, and lineage reprogramming. Small molecules not only are useful in generating functional cell types, such as cardiomyocytes, hepatocytes, pancreatic β cells, and neurons, but also can provide a better understanding of the detailed mechanisms underlying specific reprogramming processes

In this review, we will focus on making iPSCs, neurons, cardiomyocytes, hepatocytes and pancreatic β cells by small molecules (Fig. 1). Many interesting small molecules have been identified that can significantly promote cellular reprogramming (Table 1). The rapid and exciting advances in cellular reprogramming by small molecules will undoubtedly advance biomedical studies and clinical translation.

**Table 1:** Representative small molecules for reprogramming cell fates

Name	Chemical formula	Function	Application
5-Azacytidine (5-aza)		DNA methyltransferase inhibitor	iPSC reprogramming
5-Aza-2’-deoxycitidine (5-aza-dc)		DNA methyltransferase inhibitor	iPSC reprogramming
RepSox (E616452)		ALK5 inhibitor	iPSC reprogramming
Kenpaullone		GSK3β inhibitor, CDK inhibitor	iPSC reprogramming
BIX01294		G9a inhibitor	iPSC, iCM, pancreatic β-like cell reprogramming
Bayk8644		L-type Ca^2+^ channel agonist	iPSC and pancreatic β-like cell reprogramming
RG108		DNA methyltransferase inhibitor	iPSC, iNSC, pancreatic β-like cell reprogramming
CHIR99021		GSK3β inhibitor	iPSC, iN, iNSC, iCM, iCPC, iHep reprogramming
Tranylcypromine (Parnate)		Lysine-specific demethylase 1 inhibitor	iPSC,iNSC, iCM reprogramming
Valproic acid (VPA)		Histone deacetylase inhibitor	iPSC, iNSC, iCM reprogramming
AMI-5		Protein arginine methyltransferase inhibitor	iPSC reprogramming
A83-01		TGFβ signaling pathway inhibitor	iPSC, iNSC, iCM, iHep reprogramming
Forskolin		cAMP signaling activator	iPSC, iN, iCM reprogramming
2-Methyl-5-hydroxytryptamine (2-Me-5-HT)		5-HT receptor agonist	iPSC reprogramming
D4476		Casein kinase inhibitor	iPSC reprogramming
3-Deazaneplanocin (DZNep)		DNA methyltransferase inhibitor	iPSC reprogramming
AM580		RARα agonist	iPSC reprogramming
EPZ004777		DOT1L inhibitor	iPSC reprogramming
SGC0946		DOT1L inhibitor	iPSC reprogramming
Sodium butyrate (NaB)		Histone deacetylase inhibitor	iPSC and iNSC reprogramming
PS48		PDK1 activator	iPSC reprogramming
PD0325901		MAPK/ERK signaling pathway inhibitor	iPSC reprogramming
ISX9		Activates the expression of endogenous neurogenic transcription factors	iN reprogramming
I-BET151		BET family protein inhibitor	iN reprogramming
SP600125		JNK inhibitor	iN and iNSC reprogramming
GO6983		Pan-PKC inhibitor	iN reprogramming
Dorsomorphin		BMP signaling inhibitor	iN reprogramming
LDN193189		BMP signaling inhibitor	iN, iNSC reprogramming
SB431542		TGFβ signaling pathway inhibitor	iN, iCM, pancreatic β-like cell reprogramming
TTNPB		Synthetic retinoic acid receptor ligand	iN reprogramming
Thiazovivin (Tzv)		ROCK inhibitor	iPSC, iN reprogramming
DAPT		γ-secretase inhibitor	iN reprogramming
Smoothened agonist (SAG)		Hedgehog signaling activator	iN reprogramming
Purmorphamine (Purmo)		Hedgehog signaling activator	iN reprogramming
Lysophosphatidic acid (LPA)		GPCR activator	iNSC reprogramming
Rolipram		Phosphodiesterase-4 inhibitor	iNSC, iCM reprogramming
Retinoic acid (RA)		RAR receptor	iNSC reprogramming
Hh-Ag1.5		Hedgehog signaling activator	iNSC reprogramming
SMER28		Autophagy enhancer	iNSC reprogramming
Y-27632		ROCK1 inhibitor	iN, iCM reprogramming
JAK inhibitor I		JAK inhibitor	iCM reprogramming
LY-364947		TGFβR-I inhibitor	iCM reprogramming
SD-208		TGFβR-I inhibitor	iCM reprogramming
GW788388		ALK5 inhibitor	iCM reprogramming
ICARIIN		Exhibits a variety of pharmacological actions	iCM reprogramming
PD169316		p38 inhibitor	iCM reprogramming
AS8351		KDM5B inhibitor	iCM reprogramming
SC1		ERK1, RasGAP inhibitor	iCM reprogramming
OAC2		Oct4 activator	iCM reprogramming
SU16F		PDGFR inhibitor	iCM reprogramming
JNJ10198409		PDGFR inhibitor	iCM reprogramming
SU5402		FGFR, VEGFR, and PDGFR inhibitor	iCPC reprogramming
Compound E		Notch signaling inhibitor	iHep, pancreatic β-like cell reprogramming
Nicotinamide		Coenzyme, cofactor	Pancreatic β-cell reprogramming
5’-N-ethylcarboxamidoadeno-sine (NECA)		Adenosine receptor agonist	Pancreatic β-like cell reprogramming
BRD7552		PDX1 expression inducer	Increases insulin expression
Vitamin C		Cofactor of epigenetic modulators	Increases reprogramming efficiency

## PLURIPOTENT REPROGRAMMING

In 2006, the first generation of iPSCs were derived by using four transcription factors (Oct4, Sox2, Klf4, and c-Myc, termed OSKM) (Takahashi and Yamanaka, 2006). During the last 10 years, substantial efforts have been made to improve the efficiency and safety of iPSC reprogramming by non-integrating viral vectors, synthetic RNAs, recombinant proteins and small molecules (Gonzalez et al., 2011). Particularly, screenings have been applied to identify chemical compounds that can replace individual or multiple Yamanaka factors. Ichida et al. carried out a high-content chemical screening and identified RepSox (also named as E616452, an ALK5 inhibitor) as a chemical replacer of Sox2 (Ichida et al., 2009). The authors suggested that RepSox promoted the completion of reprogramming through induction of *Nanog*. In addition, RepSox possibly promoted mesenchymal-to-epithelial transition (MET) during the early phase of reprogramming (Li et al., 2010). Using a *Nanog*-Luciferase reporter as readout, Kenpaullone was found to functionally replace Klf4 in the presence of OSM (Lyssiotis et al., 2009). Shi et al. found that the combination of BIX01294 and Bayk8644 or BIX01294 and RG108 enabled OK-induced reprogramming of mouse fibroblasts, and Li et al. further reported successful OK-reprogramming of human somatic cells by using CHIR99021 and Tranylcypromine (also named Parnate) (Li et al., 2009; Shi et al., 2008). Huangfu et al. reported that Valproic acid (VPA) could promote OS-induced reprogramming of human fibroblasts (Huangfu et al., 2008). Taken together, these studies demonstrated that epigenetic regulators play important roles in iPSC reprogramming. Subsequently, several groups have focused on Oct4-only reprogramming by employing different chemical cocktails. Chen et al. found that bone morphogenetic proteins (BMPs) could support efficient mouse Oct4-alone reprogramming partly through the promotion of MET (Chen et al., 2011). Interestingly, Yuan et al. reported that AMI-5 and A83-01 enabled Oct4-induced reprogramming of mouse fibroblasts. Remarkably, these Oct4-only iPSCs were truly pluripotent and could give rise to live-born pups by tetraploid complementation assay (Yuan et al., 2011). Furthermore, Li et al. identified a specific chemical combination, including VPA, CHIR99021, E616452 and Tranylcypromine, which was sufficient to reprogramming mouse fibroblasts to iPSCs with Oct4 alone (Li et al., 2011). Subsequently, some interesting chemical substitutes of Oct4 in mouse iPS-reprogramming were identified, including Forskolin, D4476, and 2-Methyl-5-hydroxytryptamine (2-Me-5-HT) (Hou et al., 2013).

All chemical induced pluripotent reprogramming can provide a promising paradigm for cell fate transitions. In 2013, Hou et al. demonstrated successful reprogramming of mouse cells into iPS cells for the first time by using a novel cocktail of seven small molecules, including VPA, CHIR99021, E616452, Tranylcypromine, Forskolin, 3-deazaneplanocin A (DZNep), and TTNPB (Hou et al., 2013). Further refinement of this all-chemical reprogramming led a 1000-fold greater efficiency with additional small molecules, including AM580, EPZ004777, SGC0946, and 5-aza-2-deoxycitidine (5-aza-dC) (Zhao et al., 2015b). Interestingly, chemical induced pluripotent reprogramming process required the early formation of extra-embryonic endoderm (XEN)-like cells and a late transition from XEN-like cells to iPSCs, which fundamentally distinct from the pathway of TF-induced pluripotent reprogramming. However, it is still largely unknown how small molecules activate the endogenous gene regulation network, and gradually enable cell fate transitions. The remaining question is obvious: how to achieve chemical induced pluripotent reprogramming of human cells? We previously made some progress and reported that a chemical cocktail of Sodium butyrate (NaB), Parnate, PS48, CHIR99021, A83-01 and PD0325901 enabled OCT4-induced reprogramming of human primary somatic cells (Zhu et al., 2010). These human OCT4-only iPSCs were pluripotent and could give rise to cells of all three germ layers both *in vitro* and *in vivo*. Further mechanistic studies demonstrated that a metabolic switch from oxidative phosphorylation to glycolysis is an important step in iPSC reprogramming. More efforts are required to identify chemical substitutes for OCT4 in human cell reprogramming. The demonstration of human pluripotent reprogramming by an all-chemical approach will greatly benefit the study of stem cell biology and regenerative medicine.

## NEURAL REPROGRAMMING

An increasing number of people suffer from neurodegenerative disorders, such as Alzheimer’s disease and Parkinson’s disease, as life expectancy further extends. It is not feasible to obtain sufficient amounts of patient specific neural cells for disease modeling and drug development. In the last few years, great progress has been made using TF-based direct reprogramming towards induced neurons (iNs) or induced neural stem cells (iNSCs).

In 2010, Vierbuchen et al. firstly reported that mesodermal mouse fibroblasts could be rapidly and efficiently reprogrammed into ectodermal iNs by using three TFs: Brn2, Ascl1, and Myt1 l (BAM) (Vierbuchen et al., 2010). Subsequently, BAM plus NEUROD1 was applied for neuronal reprogramming of human cells (Pang et al., 2011). So far, a number of groups have generated different subtypes of neurons by combining BAM with specific TFs that play an important role in the development of specific neuron subtypes (Caiazzo et al., 2011; Kim, 2011; Pang et al., 2011; Son et al., 2011). Besides TFs, microRNAs, such as miR-9 and miR-124, can also facilitate neuronal reprogramming (Ambasudhan et al., 2011; Yang et al., 2016; Yoo et al., 2011). Obviously, it is desirable to develop non-viral and non-integrative iN reprogramming approaches by small molecules. In 2015, two simultaneous articles reported the successful generation of iN from mouse and human fibroblasts via chemical-only approaches (Hu et al., 2015; Li et al., 2015). Li et al. showed that mouse fibroblasts could be converted into neurons by using an optimal cocktail of four small molecules (Forskolin, ISX9, CHIR99021, and I-BET151, termed FICB) (Li et al., 2015). The authors suggested that I-BET151, a BET family protein inhibitor, suppressed the fibroblast-specific program, and ISX9 activated the expression of endogenous neurogenic transcription factors, which synergistically promoted neuronal conversion (Li et al., 2015). Hu et al. demonstrated the generation of neurons from human fibroblasts using a small molecule cocktail (VPA, CHIR99021, RepSox, Forskolin, SP600125, GO6983, Y-27631, and Dorsomorphin) (Hu et al., 2015). Furthermore, via this chemical cocktail, fibroblasts from familial Alzheimer’s disease patients could also be reprogrammed to iNs. Such cells can be used for *in vitro* disease modeling and drug screenings (Hu et al., 2015). Subsequently, Zhang et al. reported that sequential addition of a cocktail of small molecules (LDN193189, SB431542, TTNPB, Thiazovivin (Tzv), CHIR99021, VPA, DAPT, Smoothened agonist (SAG), and Purmorphamine) can reprogram human astrocytes into functional neurons (Zhang et al., 2015). Mechanistically, these small molecules inhibited glial but activated neuronal signaling pathways through epigenetic and transcriptional modulation. Remarkably, these human iNs were functional and could survive more than 5 months under cell culture conditions.

Compared with neurons, expandable and multipotent iNSCs are desirable for downstream applications, like disease modeling and drug screening. In the past few years, many groups reported the generation of iNSCs using neural lineage-specific TFs. These iNSCs are multipotent and can differentiate into functional neurons, astrocytes, and oligodendrocytes both *in vitro* and *in vivo* (Ring et al., 2012; Zhou and Tripathi, 2012). Recently, we achieved both mouse and human iNSC reprogramming by a cell-activation signaling-directed (CASD) strategy (Kim et al., 2011; Zhu et al., 2015). The CASD strategy uses transient exposure of somatic cells to reprogramming factors (Oct4, Sox2, Klf4, and c-Myc) in conjunction with soluble lineage-specific signals to reprogram cells into other cell types, such as iNSCs. Several interesting small molecules could promote OCT4-mediated iNSC reprogramming process, including A83-01, CHIR99021, NaB, Lysophosphatidic acid (LPA), Rolipram and SP600125 (Zhu et al., 2014a). Furthermore, similar to iN reprogramming, there are also great advances in iNSC reprogramming by using small molecules alone. In 2014, Cheng and colleagues used three small molecules VPA, CHIR99021, and RepSox to derive iNPCs from somatic cells (Cheng et al., 2014). More recently, Zhang et al. achieved more efficient mouse iNSC reprogramming by using a cocktail of nine components (CHIR99021, LDN193189, A83-01, Retinoic acid (RA), Hh-Ag1.5, RG108, Parnate, SMER28, and bFGF) (Zhang et al., 2016a). They provided definitive evidence that these iNSCs could be reprogrammed from fibroblasts using a genetic lineage-tracing system. Interestingly, further mechanistic studies uncovered that these small molecules could gradually and specifically activate key neurogenic regulators, such as Sox2, and then facilitated the neural cell fate transition.

Direct *in vivo* reprogramming will provide a perspective for cell-based clinical regenerative therapy (Chen et al., 2015; Li and Chen, 2016). Glial cells are the most abundant cells in adult brains and several groups have reported the successful TF-based reprogramming of glial cells to neurons or iNPCs. Niu et al. found that delivery of Sox2 could reprogram endogenous astrocytes to proliferating neuroblasts and these neuroblasts further differentiated to functional neurons that integrated into neural networks in the brain (Niu et al., 2013). Guo et al. demonstrated that cortical glial cells activated by injury or disease could be reprogrammed by NeuroD1 *in vivo* (Guo et al., 2014). The further application of knowledge learned from *in vitro* chemical screening and ambitious *in vivo* chemical screening will undoubtedly advance this field.

## CARDIAC REPROGRAMMING

The adult mammalian heart possesses little regenerative capacity following injury. Cardiac fibroblasts account for a majority of cells in the heart, and cardiac reprogramming holds great potentials. In 2010, Ieda et al. reported that postnatal cardiac fibroblasts could be directly reprogrammed into induced cardiomyocyte-like cells (iCMs) by transfection with a combination of three TFs (Gata4, Mef2c, Tbx5, termed GMT) (Ieda et al., 2010). Lineage-tracing experiments showed that the cardiac reprogramming with GMT was a direct conversion process. Subsequently, other groups showed that addition of TFs such as Hand2 and Nkx2.5 to GMT promoted the reprogramming efficiency or maturation of iCMs (Addis and Epstein, 2013; Ifkovits et al., 2014; Song et al., 2012). Additionally,miRNAs, such as miR-1 and miR-133, also play important roles in cardiac reprogramming (Ieda, 2016; Jayawardena et al., 2012; Muraoka et al., 2014; Nam et al., 2013; Zhao et al., 2015a). Although the efficiency of cardiac reprogramming has been improved in recent years, the molecular mechanisms of this process are largely unknown. More recently, Zhou et al. carried out a small-scale functional screening and identified that loss of Bmi1 significantly promoted mouse cardiac reprogramming. Mechanistically, Bmi1 blocked cardiac reprogramming through direct interactions with the regulatory regions of many cardiogenic genes (Zhou et al., 2016). Compared with mouse cardiac reprogramming, human cardiac reprogramming is much more challenging to be achieved. Fu et al. showed that GMT plus ESRRG, MESP1, Myocardin, and ZFPM2 could reprogram human fibroblasts to iCMs (Fu et al., 2013). Nam et al. demonstrated that human fibroblasts could be converted to iCMs by introduction of GATA4, HAND2, TBX5, Myocardin, miR-1, and miR-133 under long-term culture (Nam et al., 2013). Wada et al. found that GMT plus MESP1 and Myocardin could reprogram human fibroblasts to iCMs (Rie et al., 2013). Such TF-reprogrammed iCMs exhibited sarcomere formation, calcium transients, and action potentials, but could not contract spontaneously and robustly. These results indicated that further improvements are still required for complete human cardiac reprogramming.

Small molecules can potentially replace TFs and provide a novel approach for cardiac reprogramming. Interestingly, the addition of TGF-β inhibitor SB431542 or A83-01 improved the efficiency of iCM generation (Fu et al., 2013; Zhao et al., 2015a). Other small molecules, including Y-27632, JAK inhibitor I, LY-364947, SD-208 and GW788388, did also enhance cardiac reprogramming (Lalit et al., 2016; Zhao et al., 2015a, b). Tamakawa et al. showed that adding fibroblast growth factor (FGF) 2, FGF10, and vascular endothelial growth factor (VEGF) to GMT enhanced cardiac reprogramming about 100-fold compared with GMT only (Yamakawa et al., 2015). Wang et al. identified a small molecule cocktail, including CHIR99021, SB431542, Parnate, and Forskolin, that enabled efficient conversion of mouse fibroblasts into iCMs with Oct4 alone employing the CASD paradigm. These iCMs spontaneously contracted and exhibited a ventricular phenotype (Wang et al., 2014). Recently, there have been significant achievements in cardiac reprogramming by using small molecules alone. Fu et al. showed that a chemical cocktail (CHIR99021, RepSox, Forskolin, and VPA) could induce beating clusters of cardiac cells from mouse fibroblasts. Other small molecules, including ICARIIN, PD169316 and Rolipram also increased the efficiency of cardiac reprogramming (Fu et al., 2015). Nan et al. showed that human iCMs could be generated from fibroblasts by a specific set of small molecules (Cao et al., 2016). This combination of nine compounds was termed 9C, including CHIR99021, A83-01, BIX01294, AS8351, SC1, Y-27632, OAC2, SU16F, and JNJ10198409. Remarkably, at both transcriptome and epigenetic levels, these human iCMs resembled human cardiomyocytes. The authors observed enrichment of H3K4me3 as well as H3K27ac, and decrease of H3K27me3 on a cohort of heart developmental genes during 9C-induced cardiac reprogramming. Mechanistically, 9C treatment epigenetically and transcriptionally activated key cardiac developmental genes and facilitated the cardiac fate transition. Interestingly, unlike human iCMs from TF-based reprogramming, these human 9C-induced iCMs uniformly contracted. More efforts are required to apply this chemical cardiac reprogramming approach to patient cells.

Expandable and multipotent induced cardiac progenitor cells (iCPCs) are more suitable for basic studies and translational applications. Islas et al. found that human dermal fibroblasts could be converted into cardiac progenitors by ETS2 and MESP1 overexpression (Islas et al., 2012). After a screening of multiple cardiac TFs, Lalit et al. reported that a combination of Mesp1, Tbx5, Gata4, Nkx2.5, and Baf60c could reprogram mouse fibroblasts into iCPCs (Lalit et al., 2016). Meanwhile, Zhang et al. demonstrated that fibroblasts could be induced into expandable iCPCs by the CASD lineage conversion strategy and these iCPCs could be further long-term expanded using a combination of growth factors and small molecules (BMP4, Activin A, CHIR99021 and SU5402) (Zhang et al., 2016b). The activation of Wnt signaling by CHIR99021 not only promoted iCPC proliferation, but also inhibited further differentiation of iCPCs into cardiomyocytes. In addition, treating iCPC with Wnt inhibitor IWP2 facilitated even more efficient cardiac differentiation. Reprogramming mouse and human fibroblasts into expandable cardiac progenitors by small molecules alone could likely be achieved with further screening and modifications in the future.

Cardiac fibroblasts account for a majority of cells in the heart and represent a potential cell source for cardiac reprogramming. Direct *in vivo* cardiac reprogramming has already demonstrated some promising results. Qian et al. converted mouse resident non-myocytes into iCMs *in vivo* by local delivery of GMT after coronary ligation (Qian et al., 2012). Song et al. showed that GMT plus Hand2 could cooperatively reprogram dividing non-cardiomyocytes into functional iCMs in situ (Song et al., 2012). These cardiac reprogramming TF-delivered hearts had decreased infarct size and better cardiac function. Therefore, reprogramming cardiac fibroblasts to iCMs in their native environment by chemical compounds will hold great potentials in cardiac regenerative therapies.

## HEPATIC REPROGRAMMING

The liver is a fascinating organ that can regenerate itself; but unfortunately, this remarkable capacity is lost in chronic liver diseases. Currently, there are over 600 million people with liver diseases and 1 million deaths per year worldwide. Because the limitation of organ donors, cell-based therapy may represent an attractive approach for treating life-threatening liver failure, if a sufficient amount of functional hepatocytes can be generated from easily accessible cell sources. Many research groups focus their efforts on the generation of induced hepatocyte-like cells (iHeps) via reprogramming approaches. In 2011, Huang et al. showed that functional iHep cells could be converted from p19 null mouse fibroblasts via transduction of Gata4, Hnf1α, and Foxa3 (Huang et al., 2011). Moreover, these iHep cells restored liver functions *in vivo* and rescued the recipients from death, which provided a promising proof of principle example for the use in regenerative medicine. Meanwhile, Sekiya et al. converted mouse embryonic and adult fibroblasts into iHep cells using a combination of Hnf4α plus Foxa1, Foxa2 or Foxa3 (Sekiya and Suzuki, 2011). Remarkably, after overexpressing Hnf1β and Foxa3, mouse embryonic fibroblasts were converted to induced hepatic stem cells, which had the capability of long-term expansion and bipotential differentiation to hepatocytes and cholangiocytes (Yu et al., 2013). Subsequently, human fibroblasts were successfully reprogrammed into iHep cells by different combinations of TFs (Du et al., 2014; Huang et al., 2014). Huang et al. generated human iHeps from fibroblasts by introduction of FOXA3, HNF1α, and HNF4α (Huang et al., 2014). Meanwhile, Du et al. showed that human fibroblasts could be reprogrammed to functional iHeps by overexpressing HNF1α, HNF4α, HNF6, ATF5, PROX1, and CEBPα (Du et al., 2014). These human iHeps expressed liver-specific markers, and could integrate into mouse liver and demonstrated many metabolic functions of the liver.

Recently, the non-viral and non-integrating transduction systems, including synthetic RNAs, recombinant proteins, and episomal plasmids, become more attractive (Fusaki et al., 2009; Kim et al., 2009, 2015; Warren et al., 2010; Zhou and Freed, 2009). For example, Simeonov et al. used a method of repeating transfection with synthetic modified mRNAs encoding hepatic reprogramming TFs and successfully induced iHep cells from human fibroblasts (Simeonov and Uppal, 2014). Kim et al. applied an oriP/EBNA1-based episomal system to deliver a set of transcription factors, including Gata4, Hnf1α, and Foxa3, to convert fibroblasts into iHep cells (Kim et al., 2015). Small molecules also played a critical role in iHep reprogramming process (Lim et al., 2016; Pournasr et al., 2015). We previously generated induced endodermal progenitor cells by the CASD lineage conversion strategy, and greatly expanded these endodermal progenitor cells by a chemically defined condition, consisting of EGF, bFGF, A83-01, and CHIR99021. Further treatment with soluble growth factors and small molecules, such as A83-01 and Compound E, these induced endodermal progenitor cells efficiently differentiated into functional hepatocytes. After transplantation, human iHeps integrated into immunodeficient mouse livers, expanded extensively, and acquired mature hepatocyte functions (Zhu et al., 2014b). Moreover, Lim et al. reported that direct conversion into iHeps is a stepwise transition involving the sequential erasure of somatic memory, MET transition, and induction of hepatic cell fate (Lim et al., 2016). Through chemical screening, they found that CHIR99021 and A83-01 facilitated one factor Hnf1α-induced hepatic reprogramming. Mechanistically, these small molecules facilitated the robust iHep generation through promoting MET process. In the future, utilizing all-chemical method to induce iHep reprogramming will be a promising way to promote the development of this field.

More recently, *in vivo* hepatic reprogramming was fulfilled by injection of hepatic reprogramming factors *in situ* (Rezvani et al., 2016; Song et al., 2016). Rezvani et al. developed *in vivo* reprogramming of myofibroblasts into hepatocytes using adeno-associated virus (AAV) vectors expressing hepatic transcription factors: *Foxa1*, *Foxa2*, *Foxa3*, *Gata4*, *Hnf1a*, and *Hnf4a*. These iHeps converted from myofibroblsts were functional and reduced liver fibrosis (Rezvani et al., 2016). Song et al. showed that overexpression of *FOXA3*, *GATA4*, *HNF1A*, and *HNF4A* could convert mouse myofibroblasts into iHeps cells in fibrotic mouse livers and also reduced liver fibrosis (Song et al., 2016). More efforts are required to develop better delivery methods and improve the conversion efficiency and cell maturity. Obviously, *in vivo* hepatic reprogramming by small molecules will facilitate the translation of the iHep technology into clinical treatment of chronic liver diseases.

## PANCREATIC REPROGRAMMING

Diabetes mellitus represents a global health epidemic and affects more than 300 million people worldwide according to the International Diabetes Federation. Diabetes is caused and developed as a deficiency and/or dysfunction of pancreatic β cells. A key method to study diabetes and treat patients is to obtain unlimited numbers of functional pancreatic β cells. Reprogramming other types of somatic cells to pancreatic β cells is under active investigation. In 2005, Minammi et al. reported that adult mouse pancreatic exocrine cells could be converted into insulin-producing cells *in vitro* by suspension culture with EGF and nicotinamide (Minami et al., 2005). In 2008, Zhou et al. reprogrammed adult pancreatic exocrine cells to β-like cells *in vivo* by introducing three TFs: Pdx1, Ngn3, and Mafa (Zhou et al., 2008). Then, Li et al. developed an improved method for *in vivo* pancreatic conversion (Li et al., 2014b). These induced pancreatic β-like cells formed the islet-like structures and could persist *in vivo* for more than one year (Li et al., 2014a). Additionally, Chen et al. reported that transient intestinal expression of Pdx1, Ngn3, and Mafa promoted rapid conversion of intestinal crypt cells into endocrine cells (Chen et al., 2014). Besides mouse cells, human pancreatic ductal cells could also be converted into pancreatic β-like cells by overexpression of PDX1, NGN3, MAFA, and PAX6 (Lee et al., 2013). These converted pancreatic β-like cells could secrete insulin in response to high concentration of glucose. More interestingly, Ariyachet et al. reported that cells of the antral stomach were ideal cell source for pancreatic reprogramming (Ariyachet et al., 2016). These reprogrammed insulin^+^ β-like cells performed molecularly and functionally similar to bona fide pancreatic β cells. These results suggested that the initial cell type can affect the reprogramming efficiency and induced cell phenotype and its functional properties.

Introducing exogenous genetic material and altering the genome raise safety concerns (Barrilleaux and Knoepfler, 2011). Therefore, using small molecules to active endogenous TFs is an attractive method for cellular reprogramming. By high-throughput screening, BRD7552 was shown to be an inducer of *PDX1* expression (Yuan et al., 2013). In another report, DNA methyltransferase inhibitor 5-aza-dC was capable of increasing the expression of *Ngn3* in PANC-1 cells (Lefebvre et al., 2010). In our previous study, we found that several specific small molecules could enhance the endodermal conversion process, including CHIR99021, NaB, Parnate, RG108, and 5’-N-ethylcarboxamidoadenosine, and promote pancreatic β-like cell differentiation and maturation, including Compound E, Vitamin C, and Bayk8644 (Zhu et al., 2016). More recently, Wang et al. described the derivation of human induced endodermal progenitor cells from gastrointestinal epithelial cells using a cocktail of defined small molecules, including Bayk8644, BIX01294, RG108, and SB431542, along with support from tissue-specific mesenchymal feeders (Wang et al., 2016). Induced endodermal progenitor cells could subsequently differentiate into more specified cell types, including pancreatic β-like cells. However, the therapeutic potentials of these pancreatic β-like cells have not been investigated so far. Obviously, using small molecules to replace master TFs as well as promote reprogramming to mature pancreatic β cells is a promising therapy for treating diabetes. However, transferring these findings for efficient *in vivo* pancreatic reprogramming, preferably be an all-chemical approach is waiting to be achieved in the future.

## PERSPECTIVE

**Figure 2:**
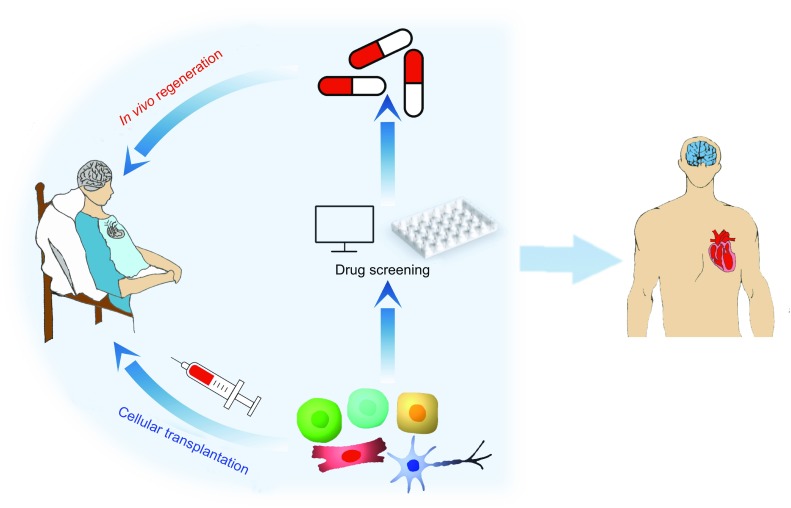
**Potential applications of cellular reprogramming**. Cellular reprogramming can provide a large number of functional cells, which can be used for cell-based transplantation and high-throughput chemical screenings. This technology will help develop drugs to stimulate patients’ endogenous cells to repair and regenerate *in vivo* in the near future. Cellular reprogramming by only small molecules will significantly advance biomedical studies and clinical applications, and realize the long-term goal of curing degenerative diseases, injuries, and aging

Cell fate transition and regeneration have fascinated biologists for centuries. After the discovery of iPSC technology, a lot of progress has been made in TF-mediated cellular reprogramming into many different defined cell types. Compared with the introduction of TFs by viral methods, small molecules have several advantages, can enhance reprogramming efficiency and improve the quality of the reprogrammed cells. Currently, identification of novel and effective small molecules for cellular reprogramming remains very labor intense. New technologies, such as high-throughput screening (HTS2) platform (Li et al., 2012) and reporter systems generated by CRISPR tools (Hockemeyer and Jaenisch, 2016), can be applied to this field and will accelerate the discovery process. In addition, the molecular mechanisms of most reprogramming processes are largely unknown, and how TFs or small molecules mediate cell fate transitions is still unclear. New technologies, such as single-cell analysis (Wen and Tang, 2016) and CRISPR-based genome-wide screening (Shalem et al., 2014), can provide new insights and help us to better understand the underlying molecular mechanisms. A better understanding of the detailed mechanisms during reprogramming processes can also help to improve the reprogramming efficiency and acquire mature cells with complete functionality for disease modeling, drug development, and cell-based transplantation (Fig. 2). Optimistically, future studies in cellular reprogramming by small molecules will overcome current hurdles, generate new discoveries, and benefit human health.
